# Contact angle measurement of free-standing square-millimeter single-layer graphene

**DOI:** 10.1038/s41467-018-06608-0

**Published:** 2018-10-10

**Authors:** Anna V. Prydatko, Liubov A. Belyaeva, Lin Jiang, Lia M. C. Lima, Grégory F. Schneider

**Affiliations:** Faculty of Science, Leiden Institute of Chemistry, Leiden University, Einsteinweg 55, 2333CC Leiden, The Netherlands

## Abstract

Square millimeters of free-standing graphene do not exist per se because of thermal fluctuations in two-dimensional crystals and their tendency to collapse during the detachment from the substrate. Here we form millimeter-scale freely suspended graphene by injecting an air bubble underneath a graphene monolayer floating at the water–air interface, which allowed us to measure the contact angle on fully free-standing non-contaminated graphene. A captive bubble measurement shows that free-standing clean graphene is hydrophilic with a contact angle of 42° ± 3°. The proposed design provides a simple tool to probe and explore the wettability of two-dimensional materials in free-standing geometries and will expand our perception of two-dimensional materials technologies from microscopic to now millimeter scales.

## Abstract (web summary)

Experimental determination of the contact angle of a two-dimensional film is crucial to understand its wettability characteristics. Here, the authors use the captive bubble method to estimate a contact angle value of 42° ± 3° for a monolayer graphene film.

## Introduction

The wetting properties of graphene have been a subject of intensive theoretical and experimental investigations over the last decade. Extremely thin and electrically conductive, graphene is widely used in biosensors, lab-on-a-chip and microfluidics platforms where graphene is in contact with water, vapor and analytes^1–4^. Although graphene was long believed to be a graphite-like material^5–7^, some recent studies have shown a wide spread of water contact angle (CA) on graphene^5,6,8–10^, with values ranging from 10° when supported by water^10^ to 127° on solid substrates^6^. One reason for such discrepancies in the values of the contact angle is the difference in sample preparation and measurement conditions^5,8^. The adsorption of airborne hydrocarbons, the cleanliness and quality of the graphene–substrate and graphene–water interface can have significant effects on the measured contact angles, which, however, can be minimized in most cases by conducting experiments in controlled atmospheres and by avoiding the use of polymers during the transfer process^11–14^.

The wetting characteristics of a material are dictated by both the surface and the bulk properties of the material, which implies the impossibility to determine the intrinsic wetting properties of two-dimensional (2D) materials which have no bulk. In other words, all wetting characteristics of graphene, such as contact angle and surface energy, refer not only to the graphene surface but also to the bulk phase underneath it and must not be regarded as solely graphene’s properties.

In this respect, probing the wetting characteristics of free-standing graphene can give an indispensable insight for understanding graphene’s wettability. Yet, due to the extreme fragility of graphene and other 2D materials, studies on free-standing graphene have been limited to theoretical predictions with only a few experimental works on partially suspended graphene^15,16^. Being the only experimental indication of free-standing graphene’s wettability up to now, the contact angle of partially suspended graphene is still an indirect measure and requires multistep sample preparation which may result in an ill-defined graphene–substrate interface yielding a range of contact angle values.

In this work, we present a simple and clean captive bubble design for measuring directly the wettability of free-standing graphene. The captive bubble method, i.e., the injection of an air bubble underneath graphene floating on water, allows for the formation of a graphene free-standing area as large as 1.5 by 1.5 mm, the largest free-standing area that has been reported so far for a 2D material. Essentially, graphene remains floating on the water surface after copper etching, intrinsically preventing any transfer- or handling-related contamination and corrugation. An additional advantage is that the graphene side on which the contact angle is measured (i.e., the side that initially faces copper and then water) has never been exposed to ambient air and is therefore not subjected to airborne hydrocarbons adsorption^16,17,18^.

## RESULTS

### Captive bubble versus sessile drop

**Fig. 1:**
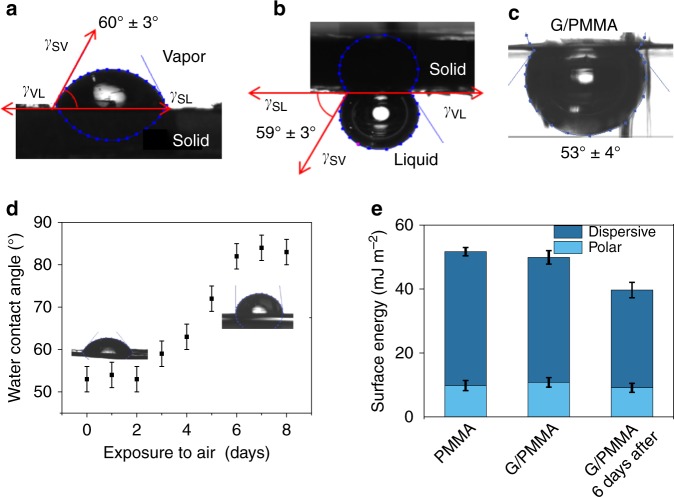
Sessile drop and captive bubble measurements of graphite and supported graphene. **a** Sessile drop of water on freshly exfoliated highly oriented pyrolytic graphite (HOPG) in air, where γ_VL_, γ_SV_, and γ_SL_ are the liquid−vapor, solid−vapor, and thesolid−liquid interfacial tensions, respectively. The measured contact angle is 60° ± 3°. The measurement was reproduced on ten samples and the error bar represents the standard deviation. **b** Captive bubble configuration on freshly exfoliated HOPG in water. The measured contact angle is 59° ± 3°. The measurement was reproduced on ten samples and the error bar represents the standard deviation. **c** Captive bubble measurement of water contact angle on graphene supported by a poly(methyl methacrylate) (PMMA) layer. The measurement was reproduced on ten samples and the error bar represents the standard deviation. **d** Sessile drop contact angle measurement of graphene supported by a PMMA layer constantly exposed to air as a function of air exposure time. The measurement was reproduced on three samples and the error bar represents the standard deviation. **e** Surface energies and polar and dispersive components of the surface energy for PMMA, graphene supported by PMMA, and graphene supported by PMMA which was exposed to air for six days. The measurement was reproduced on three samples and the error bar represents the standard deviation

The captive bubble method measures the wetting contact angle using an air bubble at a solid–liquid interface. Often, the method works best for hydrophilic substrates in which liquid spreads out yielding more difficulties to determine the contact angle with the sessile drop technique, e.g., for contact lenses and hydrogels^19,20^. The captive bubble and sessile drop configurations represent the same three-phase equilibrium and, therefore, are equivalent (Fig. 1a, b).

The difficulty of the contact angle measurement on free-standing graphene is that 2D materials do not withstand the mechanical disturbances originating from—for example—depositing a droplet of liquid on their surface because of their extreme thinness^10^. Additionally, free-standing graphene as big as a macroscopic droplet does not exist. Instead, using the captive bubble geometry (i.e., a water–graphene–air bubble interface), allows for a reliable contact angle measurement. Advantages of this method in comparison with the sessile drop technique is that deionized water is primarily composed of water molecules (and therefore less contamination per volume percent compared to air and vacuum; i.e., water protects graphene from airborne hydrocarbon contamination). Another remarkable advantage of the technique is that the bubble is saturated with water, therefore yielding a contact angle in equilibrium in time.

For the comparison of the captive bubble method with the sessile drop technique the water contact angle was measured on highly oriented pyrolytic graphite (HOPG). HOPG was exfoliated with the scotch tape in air or in water depending on the method of contact angle measurement. The average contact angles are 59° ± 3° for the sessile drop method and 60° ± 3° for the captive bubble method (Fig. 1a, b). Both methods show high repeatability on solid substrates.

Additionally, contact angles of graphene with a 300 nm layer of poly(methyl methacrylate) (PMMA) were measured using the captive bubble method and the sessile drop technique as control tests respectively. Graphene appeared wetting transparent in both cases displaying the contact angles of the bare PMMA support—that is, 53° ± 4° measured by the captive bubble method (Fig. 1c) and 54° ± 3° using the sessile drop method (Fig. 1d). Noteworthy, after two days the graphene/PMMA sample became more hydrophobic and after six days the contact angle of graphene increased up to 85° (Fig. 1d). Such transition from a hydrophilic to a hydrophobic surface is known to be caused by the adsorption of hydrocarbons from the air^11^. A surface-energy analysis using the Owens–Wendt method (Supplementary Note 1) showed that while the graphene surface is clean, hydrocarbons tend to adsorb to minimize the free surface energy. The decrease of the total surface energy and its dispersive component is consistent with previous reports (Fig. 1e)^21^.

The agreement between the sessile drop and captive bubble results for freshly exfoliated graphite and graphene/PMMA in which graphene was not exposed to air shows that, although the air in the bubble may contain hydrocarbon contaminants, they do not affect the contact angle because of the short-lived graphene-bubble contact and/or negligible amount of hydrocarbons present in the bubble.

### Captive bubble method to study graphene: inflection of floating graphene

**Fig. 2:**
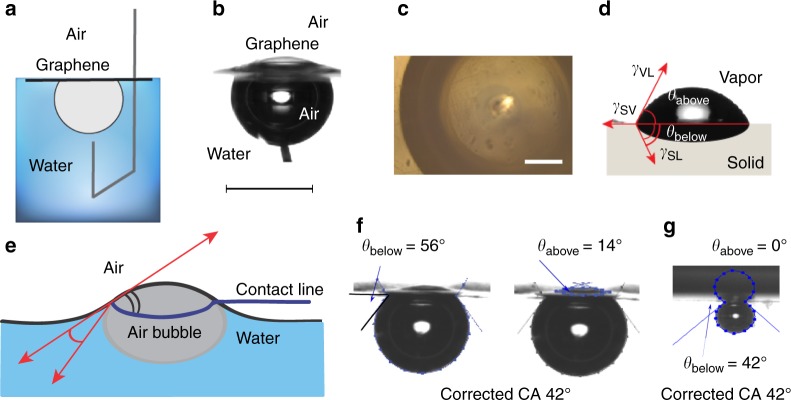
Captive bubble configuration for measuring the contact angle of water on free-standing graphene. **a** Schematic illustration of the captive bubble setup for measuring the contact angle of water on free-standing graphene. **b** Optical image of graphene on top of an air bubble (side view). Scale bar represents 2 mm. **c** Optical image of graphene suspended above the air bubble (top view). Scale bar represents 500 µm. **d** Geometry of the contact line on a soft elastic substrate. The contact angle of three phases is a sum of angles below (*θ*_below_) and above (*θ*_above_) the horizontal line. **e** Neumann’s triangle. Surface-energy balance for captive bubble on graphene. **f** Optical images of a captive bubble on graphene and calculation of the contact angle for an inflected graphene with an air bubble of 6 µl. **g** Optical image of a captive bubble on graphene and calculation of the contact angle (bubble volume 0.2 µl)

For contact angle measurement on graphene using the captive bubble technique, an air bubble is deposited using an inverted needle underneath graphene (Fig. 2a, b; for technical details on the sample preparation and contact angle measurements see the section Methods). From the optical image, one can see that the area of graphene surrounded by air on both sides is 1.5 by 1.5 mm large, the largest free-standing graphene area ever reported (Fig. 2c).

However, due to its extreme flexibility and thinness, graphene inflects above the surface of water under the pressure of the air bubble and the inflection should be taken into account for the calculation of the contact angle. For flexible materials, forces at the three-phase contact line cannot be described by the Young equation, as it is for flat rigid substrates. Instead, numerous investigations of the contact angle show that the force balance on soft materials is best described by the Neumann’s triangle^22–28^. According to Neumann’s theory the total contact angle on a deformed substrate can be described as a sum of two angles, beneath and above the contact line, i.e., *θ*_above_ + *θ*_below_ (Fig. 2d). Since the angle measured using the captive bubble method is the contact angle between the air bubble and the solid, i.e., *θ*_air_, the water contact angle should be recalculated as 180° − *θ*_air_. Taking into consideration the inflection of graphene, the contact angle of water on deflective graphene is therefore 180° − (*θ*_above_ + *θ*_below_) (Fig. 2d).

The measurements of the contact angle of an air bubble on graphene, thus, are more complex than measuring the contact angle of a drop of water on graphene, and consist of measuring the contact angle measurement above and below the three-phase contact line. The schematic and optical images of an example of a measurement of the contact angle of water on graphene are shown in Fig. 2e and f, respectively. The results show that graphene is hydrophilic with a contact angle of water of 42° ± 7° (Fig. 2f). A video of an air bubble underneath graphene is provided in the Supplementary information (Supplementary Movie 1).

Interestingly, a smaller bubble causes a decrease of the measured angle 180° −* θ*_below_ and of the inflection angle *θ*_above_, but the difference between the two, i.e., the actual contact angle, is independent of the bubble volume and equal to 42° ± 3°: for a bubble volume of 6 µl, the resulting contact angle is 42° (i.e., the difference between the measured angle of 56° and the inflection angle of 14°), and for a bubble volume of 0.2 µl, the measured angle is 42° and there is no observable inflection to account for as the smaller bubble does not induce significant stretch in the graphene sheet (Fig. 2f, g). These observations are in agreement with other reported works and hypothesis that the size-dependence of the contact angle occurs only on rough and heterogeneous surfaces and not on smooth homogeneous surfaces like graphene^29–31^.

### Few-layer graphene and modified graphene

**Fig. 3:**
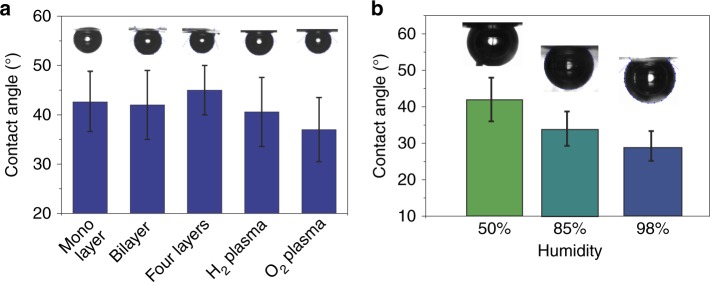
Graphene contact angles measured by the captive bubble method. **a** Contact angles of water on free-standing, monolayer, bilayer, four-layer graphene and graphene modified with H_2_ and O_2_ plasma, measured using the captive bubble method. The measurement was reproduced on five samples and the error bar represents the standard deviation. **b** Contact angle of water on free-standing graphene in 50%, 85%, and 98% relative humidity. The measurement was reproduced on two samples and the error bar represents the standard deviation

Multilayered graphene (bi- and four-layer) did not exhibit appreciable difference in the water contact angle (Fig. 3a). Since defects and chemisorption of atomic hydrogen/oxygen on graphene are known to affect wetting^32^, we also measured contact angles of graphene modified with H_2_ and O_2_ plasma (Fig. 3a, see Methods for details on plasma treatment). After modification with H_2_ plasma the contact angle on graphene-on-copper decreased from 76° ± 5° to 68° ± 5°, which can be explained by the cleaning effect of the plasma^33,34^ (Raman characterization of graphene before and after the modification, Supplementary Figure 1). We did not notice a difference in the wettability of suspended graphene after the surface modification with a H_2_ plasma. Separately, an air bubble on graphene modified by a O_2_ plasma was very unstable and tended to slip away from the graphene which could be explained by oxygen functionalities induced by the O_2_ plasma^35^. Overall, contact angle values of modified and multilayer graphene are equal to the contact angle of monolayer pristine graphene given the error margins (Fig. 3a).

### Effect of humidity

Recently graphene has been shown to turn hydrophilic when floating on water due to the wetting transparency effect^10,36^. In order to test the effect of the environment on one side of graphene on its hydrophobicity on the other side, we performed the experiments under different humidities. We measured contact angles of water using the captive bubble method with humidities of 98%, 85%, and 50% regulated by saturated salt solution of K_2_SO_4_ and KCl^37^ (see Methods for more details). Interestingly, a higher humidity level yields lower water contact angle: 29° ± 8° at 98% humidity and 42° ± 7° at 50% humidity (Fig. 3b).

As for all microscopic approaches, the captive bubble method is technically challenging for studying 2D materials because they are fragile and even small vibrations can break them apart. In some cases, cracks and holes appear on the graphene surface during the etching process^15^, which can prevent an air bubble from staying underneath the graphene. Furthermore, CVD (chemical vapor deposition) grown graphene is not monocrystalline and has grain boundaries, which could make graphene permeable to air^38,39^. In our experiments, the air bubble underneath graphene was stable from two seconds up to fifteen minutes after which either the bubble or graphene would collapse (Supplementary Figure 2 for optical images before and after the captive bubble experiment). We found that the main sources of the degradation of graphene quality when floating on water are high rate of copper etching, vibrations, intense air circulation and, partly as a result of all the aforementioned, movability of the graphene on the surface of water. In fact, the quality and stability of the floating graphene was significantly improved by using a less concentrated etchant solution of ammonium persulfate (0.3 M and lower), or by minimizing vibrations and air circulations, and, importantly, immobilizing graphene with a lipid clamp^40^ (see Methods for more details on the lipid clamp and sample preparation).

## Discussion

Although partly suspended graphene on a texturized substrate shows hydrophobic properties with contact angle up to 85°^15,16^, our findings demonstrated that clean fully free-standing graphene is mildly hydrophilic (with a measured water contact angle of 42°, in agreement with theoretical predictions on the hydrophilicity of graphene with contact angle of water ranging from 37° to 44°^11,17,41^). However, such low contact angle is rather surprising, because given the wetting transparency of graphene^9,10^, the contact angle of free-standing graphene should be identical to the contact angle of air, i.e., 180°. The wetting behavior, therefore, in this case cannot be only dictated by the transmission of water–air interactions, but is substantially affected by the phenomena occurring at the graphene surface. Remarkably, the measured contact angle values for mono-, bi-, four-layer graphene, and graphene treated with O_2_ and H_2_ plasma are similar (Fig. 3a), also supporting this assumption. The hydrophilicity of graphene (i.e., the fact that water wets free-standing graphene) could be explained by the formation of *π*-hydrogen bonding between water molecules and the aromatic system, as it is for benzene–water interaction^42,43^. Another hypotheses attributes the hydrophilicity of graphene to the spontaneous adsorption of OH^−^ ions on graphene surfaces^44^, which could lead to interactions with water and an increase of the repulsive double layer interaction between air (in the bubble) and graphene. As a complementary evidence for the hydrophilic behavior of graphene in water, stable surfactant-free dispersions of graphene have been recently obtained in degassed water^44^. The apparent inability of graphene to form stable aqueous dispersions, which was previously attributed to the hydrophobicity of graphene, is now explained by the adsorption and further coalescence of nanobubbles on the graphene surface.

On the other hand, an increase in the environment humidity, i.e., the concentration of water molecules on the top side of graphene, leads to a decreasing water contact angle value and therefore an increase in the hydrophilicity of graphene (Fig. 3b), indicating that the transparency of graphene to water–water interactions still has a substantial contribution in addition to the water–graphene interactions mentioned above.

In conclusion, we have obtained a millimeter in size-suspended 2D material by simply harvesting surface tension forces at the air–water–graphene–air interface using an air bubble captivated on graphene floating on water.

Direct contact angle measurements have shown that free-standing graphene has hydrophilic properties. Advantageously to other methods, our technique allows to probe the very water–graphene–air interface, in the cleanest way, avoiding any irregularities arising from the transfer and handling processes. The observed hydrophilicity could be explained by the formation of hydrogen bonds which would impact the spontaneous adsorption of water on the graphene surface.

We believe that this work provides a stimulus to further study the still unexplored basic properties of suspended 2D materials, as their surface chemistry, surface energy, compressive and flexural strength, and device interaction at a millimeter-scale level in a free-standing geometry.

## Methods

### Materials

Two types of graphene were used: monolayer graphene on a copper substrate provided by Graphenea and graphene grown in a tube oven on a 25 µm copper foil at 1035° according to the procedure described in ref. ^45^. Before conducting contact angle experiments, the backside of graphene-on-copper (G/Cu) was removed with a O_2_ plasma. Both types of graphene (i.e., Graphenea and homemade) showed the same results for water contact angle measurements.

Multilayered graphene was prepared by repetitive PMMA transfer^46^ of graphene on G/Cu^47,48^.

Highly oriented pyrolytic graphite (HOPG, 7 × 7 × 0.8–1.8 mm with mosaic spread 0.8–1.2 degree) was purchased from NT-MDT.

### Sample preparation

CVD graphene on a copper substrate was placed in a 0.3 M water solution of ammonium persulfate (APS) (98% Sigma-Aldrich). Once the copper foil was etched away, the APS solution was repeatedly replaced with ultrapure water by sequential diluting steps yielding a clean graphene surface without any observable APS crystals^45^. In general, the presence of ions in water has a very small effect on the surface tension of water—in the order of 3% or lower at the concentration of 0.3 M^49–51^—and, therefore, negligible effects on the measured contact angle. Consequently, and given the precautions we undertook to replace the etching APS solution by water, we assume that possible presence of residual ions had no effect on the contact angle measurements (the contact angle of water on graphene in 0.1 M FeCl_3_ is equal to the contact angle of graphene in pure water, Supplementary Figure 3).

To place a 6 µl air bubble under the water–graphene–air interface, air was injected through a J-shaped inverted needle underneath the graphene (Fig. 2a). The contact angle was then measured at least five times (unless otherwise noted) at the three-phase line interface (Fig. 2d).

To improve the stability of graphene on the water surface, graphene was surrounded with a Langmuir–Blodgett film of 1,2-dipalmitoyl-*sn*-glycero-3-phosphocholine (DPPC) lipids (Avanti Polar Lipids Inc.) at a surface pressure of 30 mN m^−1^ as it is described in refs. ^40,52^. The lipids had a concentration of 1 mg mL^−1^ and were first dissolved in CHCl_3_/CH_3_OH 3:1 vol %. First, graphene on copper^45^ (copper facing down) was placed floating on the etchant solution and the appropriate amount of lipids (depending on the size of the graphene and of the cuvette) was added on the surface of the etchant solution around graphene. The etchant solution was then sequentially replaced with ultrapure water and the contact angle was measured. The lipids are known to only spread on the surface of water (around the graphene) without adsorbing on its surface (as measured by infrared spectroscopy)^40^.

Graphene surrounded with lipids showed a higher stability during the deposition of the air bubble. Both graphene samples, without and with lipids, showed similar measured contact angles, i.e., 42° ± 3° and 42° ± 3° respectively (Supplementary Figure 4 and Supplementary Movies 1 and 2), confirming the absence of lipids on the graphene surface.

Immobilizing the graphene with lipids is essential for contact angle measurement. If graphene is not stabilized with lipids, the action of placing the air bubble creates a momentum and pushes the graphene sheet away from the field of view of the camera, despite the fact that the bubble is stable and does not collapse (Supplementary Movie 3).

For contact angle measurements of hydrogenated and oxygenated graphene, graphene was first hydrogenated (respectively, oxygenated) using a H_2_ (respectively, O_2_) plasma in a computer controlled Diener plasma generator for 247 s (1 mbar, 10 W)^34^.

### Raman spectroscopy

The quality and the number of layers of all graphene samples were characterized by Raman spectroscopy^53^ at room temperature using a 100× objective and 457 nm and 532 nm lasers at a power below 2 mW to avoid excessive thermal damage of graphene. Figure S1 displays typical Raman spectra of graphene on copper (a) and transferred onto a Si/SiO_2_ wafer (b). The shape of the 2D peak (~2700 cm^−1^), that can be fitted with a single Lorentzian component is indicative of single-layer graphene^53^. The absence of a D peak at ~1370 cm^−1^ (Figure S1a, b) suggests a low density of defects for non-treated graphene samples^53^.

For hydrogenated and oxygenated graphene, however, the appearance of the D peak (Figure S1c, d) results from the introduced *sp*^3^ defect sites^34^. Particularly, the ratio *I*(2D)/*I*(G) decreased from ~2 (pristine graphene) to ~1 after 4 min of hydrogen plasma treatment, indicating the effective doping in the lattice induced by hydrogenation^54^. Moreover, the appearance of a D′ peak (~1620 cm^−1^) in hydrogenated graphene is also related to the activation of defects. The *I*(D)/*I*(D′) value of ~10 further confirms the *sp*^3^ nature of hydrogenated defects^55^.

### Optical microscopy

Optical images of graphene on water and graphene transferred on silicon wafer were taken with a Leica optical microscope (DM 2700 M).

### Contact angle measurements

Contact angle measurements were conducted with a standard Ramé-Hart 250 goniometer (Netcong, NJ) and recorded with the DROPimage advanced v 2.8 software under ambient conditions (22 °C). Two methods were used for the characterization of wetting. For the sessile drop technique a water droplet of 5–7 μL was deposited on a substrate and contact angle was measured within five seconds. For the captive bubble method, an air bubble with a volume of 6 μL was supplied with a microsyringe at the interface with an inverted needle (28 gauge, 304 SS Ramé-Hart). The analysis of contact angles from recorded videos were made with the software ImageJ (Drop snake analysis).

### Measurements at different humidities

Experiments with controlled humidities were carried out using the saturated salt solution method, commonly used for accurate humidity control and the calibration of hygrometers^56–59^. For that, an oversaturated salt solution is placed in a closed box and certain equilibrium vapor pressure (and thus relative humidity) is created. The oversaturation of the solution assures that the built vapor pressure is stable to presence of moisture sources and sinks (the excess of the salt precipitates and the solution remains saturated with the vapor pressure unchanged) and, therefore, provides a precise humidity level. Different salts have different saturated vapor pressures at a given temperature, and the humidity thus can be varied by changing the chemical composition of the salt.

For our experiments, we used oversaturated solutions of KCl for the humidity of 85.11 ± 0.29%^37^ and K_2_SO_4_ for the humidity of 97.59 ± 0.53%^37^. For measurements at every given humidity, a beaker with the corresponding salt solution was placed in a sealed glass chamber with an embedded syringe (for further contact angle measurements) together with the cuvette containing graphene floating on water. Then the contact angle was measured using the captive bubble method. The relative humidity of 50% was the standard ambient humidity of the laboratory maintained by a moisture extractor and measured by a hygrometer, and the contact angle measurements were conducted without salt solutions.

### Surface-energy calculation

The surface energy and its components were calculated from the contact angle measurements of different liquids on target surfaces using the Owens–Wendt technique^59^. Ultrapure water, ethanol (96%), ethylene glycol (99,8%), diiodomethane (99%), and methylnaphthalene (95%) were used as test liquids. Details on measured contact angles, surface tension of liquids and surface-energy calculation are provided in the Supplementary information (Supplementary Note 1 and Supplementary Tables 1 and 2).

## Electronic supplementary material

Supplementary information

Description of Additional Supplementary Files

Supplementary Movie 1

Supplementary Movie 2

Supplementary Movie 3

## Data Availability

All data supporting the findings of this study are available within the article and its Supplementary Information. All other data are available from the corresponding author upon reasonable request.
