# Supplementary material for: Contact angle measurement of free-standing square-millimeter single-layer graphene
Source: Nat Commun. 2018 Oct 10;9:4185. doi: 10.1038/s41467-018-06608-0 (PMC6180012; doi:10.1038/s41467-018-06608-0)
Supplement: Supplementary file 1 — Supplementary information [file 41467_2018_6608_MOESM1_ESM.pdf]

SUPPLEMENTARY INFORMATION

Contact angle measurement of freestanding square millimeters of single layer graphene

Anna V. Prydatko†, Liubov A. Belyaeva†, Lin Jiang, Lia M.C. Lima and Grégory F. Schneider*

Leiden University, Faculty of Science, Leiden Institute of Chemistry, Einsteinweg 55, 2333CC Leiden, The Netherlands

†These authors contributed equally to this work.

* to whom correspondence should be addressed: g.f.schneider@chem.leidenuniv.nl

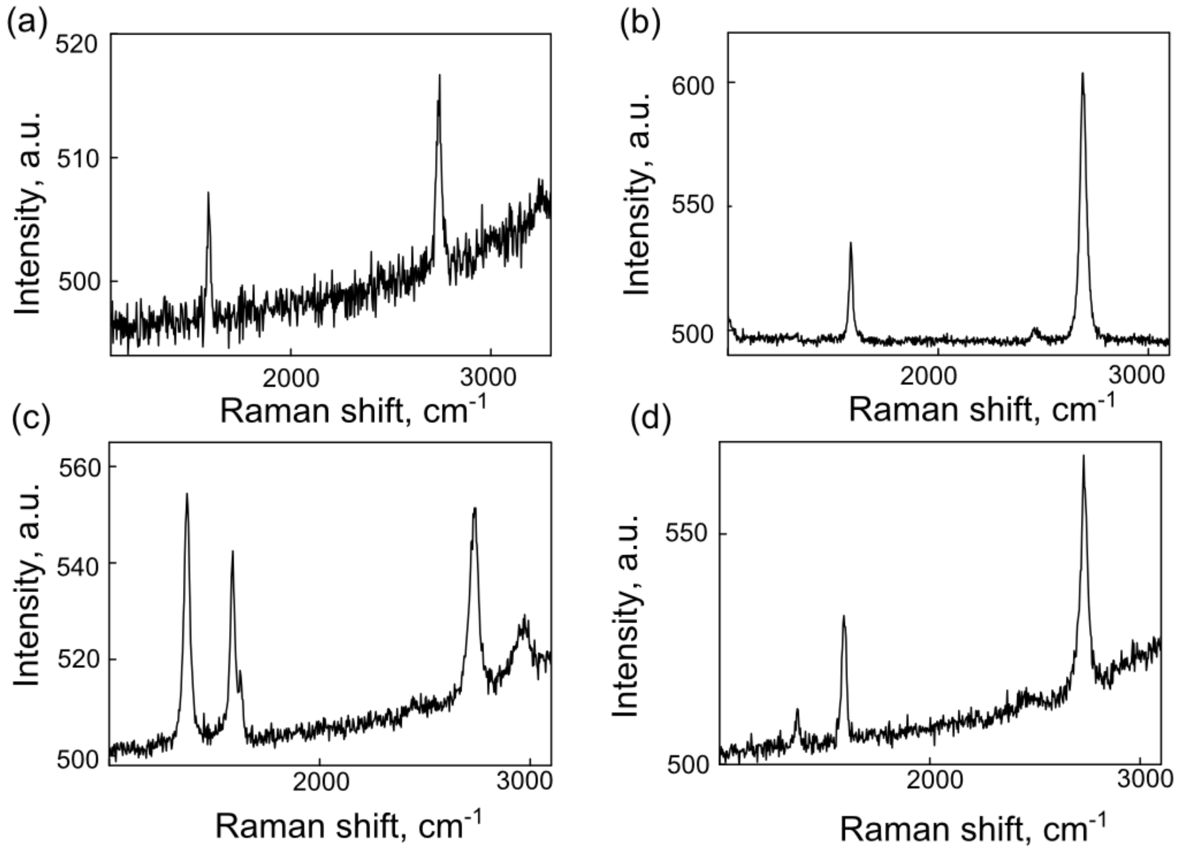

Supplementary Figure 1. Raman spectra of CVD (chemical vapor deposition) graphene before and after plasma modification. (a) Non-treated graphene on Cu after the growth. (b) Non-treated graphene transferred onto a Si/SiO_2 wafer. (c) Graphene on Cu after H_2 plasma treatment. (d) Graphene on Cu after O_2 plasma treatment.

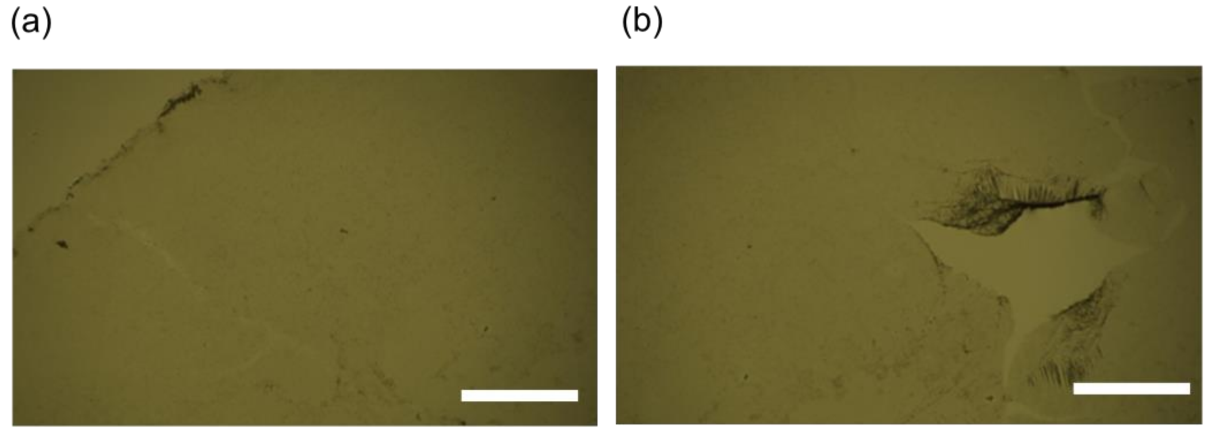

Supplementary Figure 2. Optical images of graphene floating on the surface of water. (a) Before captive bubble measurement. The scale bar represents 500 µm. (b) After captive bubble measurement. The scale bar represents 500 µm.

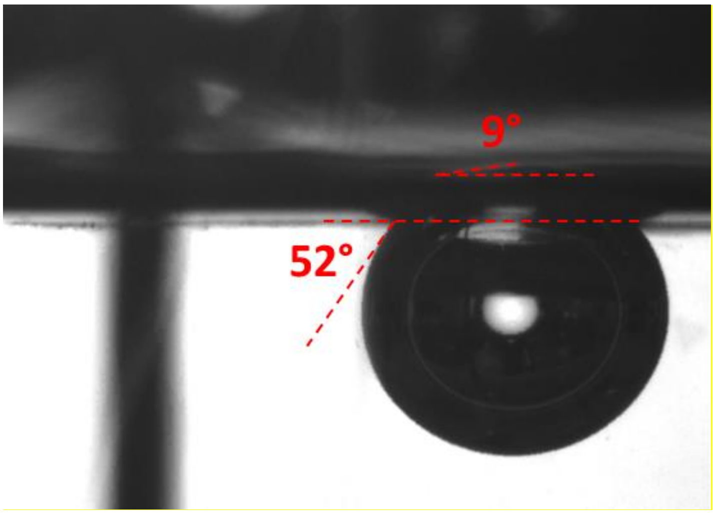

Supplementary Figure 3. Contact angle of graphene on the surface of 0.1 M aqueous solution of $FeCl_3$. Presence of ions in concentrations below 0.3 M does not affect the measured contact angle, the contact angles of graphene in different etchant solutions are equal to the contact angle of graphene in pure water.

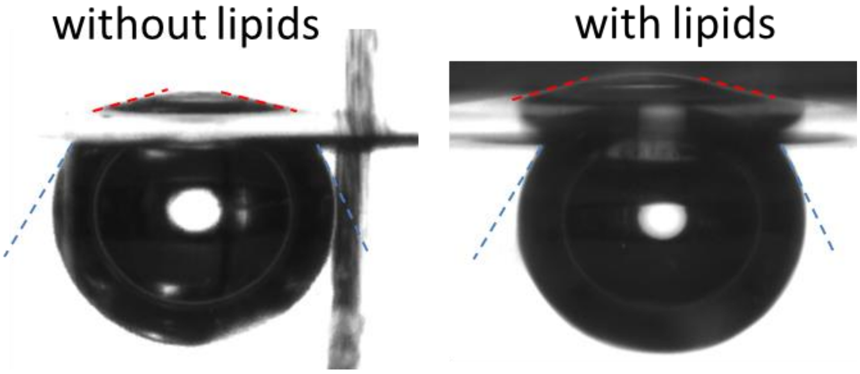

Supplementary Figure 4. Captive bubble on graphene with (right) and without (left) a DPPC (1,2-dipalmitoyl-sn-glycero-3-phosphocholine) lipid scaffold. Lipids do not affect the contact angle value of 42°±3°.

Supplementary Note 1

Calculation of surface free energy

Surface energies of bare PMMA (poly(methyl methacrylate) and PMMA-coated graphene samples were calculated according the Owens-Wendt model [1]. Based on the contact angle measurements with

liquids of different polarities, the Owens-Wendt equation allows for the determination of total surface energy of a solid and its polar and dispersive components:

$$\gamma_{\mathrm{lv}}(1+\cos\theta) = 2(\sqrt{\gamma_s^{\mathrm{d}}\gamma_l^{\mathrm{d}}} + \sqrt{\gamma_s^{\mathrm{p}}\gamma_l^{\mathrm{p}}})$$

The polar and dispersive components of liquids $\gamma_{\mathrm{L}}^{\mathrm{P}}$ and $\gamma_{\mathrm{L}}^{\mathrm{D}}$ were determined by measuring contact angles (sessile drop method) and applying the Owens/Wendt Theory for PTFE (teflon), which is a solid with known polar and dispersive components of the surface tension ($\gamma_{\mathrm{S}}{}^{\mathrm{P}}$=0 mN m^{-1}, $\gamma_{\mathrm{S}}{}^{\mathrm{D}}$=18 mN m^{-1}). The determined surface tensions and their components of all used liquids are listed in Table 1.

Then contact angles of bare and PMMA-coated graphene with the liquids listed in Table 1 were measured. The results were plotted as $\frac{\gamma_{\mathrm{L}}(cos\theta+1)}{2\sqrt{\gamma_{\mathrm{L}}^{\mathrm{D}}}}$ versus $\frac{\sqrt{\gamma_{\mathrm{L}}^{\mathrm{P}}}}{\sqrt{\gamma_{\mathrm{L}}^{\mathrm{D}}}}$ for each substrate and the dependences were fitted linearly. The slope of the plot equals $\sqrt{\gamma_{\mathrm{S}}^{\mathrm{P}}}$ and the intercept equals $\sqrt{\gamma_{\mathrm{S}}^{\mathrm{D}}}$. The squares of the latter two equal $\gamma_{\mathrm{S}}{}^{\mathrm{P}}$ and $\gamma_{\mathrm{S}}{}^{\mathrm{D}}$ respectively. The resulting surface tensions and their polar and dispersive components are presented in Supplementary Table 2 below and charted in Figure 1c of the main text.

Supplementary Table 1. Calculated surface tensions, polar and dispersive components of tested liquids

Liquid	$\gamma_{\mathrm{L}}^{\mathrm{P}}$, mJ m^{-2}	$\gamma_{\mathrm{L}}^{\mathrm{D}}$, mJ m^{-2}	$\gamma_{\mathrm{L}}^{\mathrm{total}}$, mJ m^{-2}
Water	51	21.8	72.8 ± 2.4
Ethylene glycol	19.2	28.8	48.0 ± 1.9
10% Ethanol in water	36.1	23.9	60.0 ± 2.2
Diiodomethane	0	50.8	50.8 ± 2.3
1-Methylnaphthalene	0	42.0	42.0 ± 1.1

Supplementary Table 2. Calculated surface tensions, polar and dispersive components of PMMA, freshly PMMA-coated graphene and PMMA-coated graphene aged for six days

Surface energy/Sample	PMMA	Graphene on PMMA	Graphene on PMMA after 6 days
$\gamma_S{}^P$, mN m^{-1}	9.8±1.6	10.8±1.5	9.1±1.4
$\gamma_S{}^D$, mN m^{-1}	41.9±1.3	39.1±2.1	30.6±2.4
γ_D^{total},, mN m^{-1}	51.6±2.3	47.9±1.6	39.7±1.5

Supplementary References

1. Owens, D. K. & Wendt, R. C. Estimation of the surface free energy of polymers. *J. Appl. Polym. Sci.* **13,** 1741–1747 (1969).
