# Supplementary material for: Contact angle measurement of free-standing square-millimeter single-layer graphene
Source: Nat Commun. 2018 Oct 10;9:4185. doi: 10.1038/s41467-018-06608-0 (PMC6180012; doi:10.1038/s41467-018-06608-0)
Supplement: Supplementary file 2 — Description of Additional Supplementary Files [file 41467_2018_6608_MOESM2_ESM.pdf]

Description of Additional Supplementary Files

File Name: Supplementary Movie 1
Description: Recording of an air bubble underneath graphene without lipids.

File Name: Supplementary Movie 2
Description: Recording of an air bubble underneath graphene surrounded with lipids.

File Name: Supplementary Movie 3
Description: Instability of graphene if not stabilized with lipids: an air bubble moves the graphene away from the camera field of view.
